# Applications of environmental DNA monitoring for seaweed reproductive phenology: A case study with giant kelp (*Macrocystis pyrifera*)

**DOI:** 10.1111/jpy.70000

**Published:** 2025-03-18

**Authors:** Madeline R. Ward, Christopher P. Burridge, Sharee McCammon, Adam Smolenski, Catriona L. Hurd, Wouter Visch

**Affiliations:** ^1^ Institute for Marine and Antarctic Studies University of Tasmania Hobart Tasmania Australia; ^2^ School of Natural Sciences University of Tasmania Hobart Tasmania Australia

**Keywords:** eDNA, kelp forest restoration, macroalgae, qPCR, seaweed, seaweed aquaculture, zoospores

## Abstract

Monitoring the seasonal reproductive cycles of seaweeds is crucial for effective population and ecosystem management, as well as mariculture seedstock collection. Traditional methods, such as visual monitoring by SCUBA diving or snorkeling, are costly, labor‐intensive, and limited in temporal and spatial coverage. This study explores substituting these methods with environmental DNA (eDNA) techniques for giant kelp (*Macrocystis pyrifera*, order Laminariales). This laboratory study aimed to determine the minimum detectable concentration of zoospores and sporophyte tissue needed for detecting the reproductive phenology of *M. pyrifera* and to assess the ability and sensitivity to discriminate between life stages. The study involved syringe‐filtering seawater samples through 0.45‐μm pore‐size filters before quantitative polymerase chain reaction (qPCR) analysis with species‐specific primers. There was a strong positive correlation between zoospore concentration and eDNA copies per μL (ρ = 0.982, *p* < 0.001), and a weak correlation for sporophyte wet weight (ρ = 0.367, *p* = 0.134). There was a significant difference between zoospore and zoospore + sporophyte treatments (*p* = 0.010), indicating the substantial influence of sporophyte tissue on detected eDNA quantity. Sporophyte tissue obscures the zoospore signal, especially at lower zoospore concentrations (<37 zoospores · mL^−1^), highlighting that eDNA analysis is suitable for monitoring reproductive peaks and broader patterns in seasonal reproduction cycles of giant kelp when zoospore concentrations are high.

**Abbreviations**
ANOVA: analysis of variance
Cq‐value: quantification‐cycle values
eDNA: environmental DNA
NTC: no template control
qPCR: quantitative polymerase chain reaction

## INTRODUCTION

Seaweeds are important primary producers in coastal ecosystems (Hurd et al., 2014), forming habitat and fostering ecosystem diversity (Duarte & Cebrián, 1996). They are also important in a global mariculture context (Food and Agriculture Organization [FAO], 2020) and contribute to economies by helping create healthy coastal systems by providing valuable ecosystem services, such as supporting both tourism and commercial fisheries (Bennett et al., 2015). In monetary terms, the value of ecosystem services in marine kelp forests globally is estimated to be between US$465 and US$562 billion per year (Eger et al., 2023). Despite their value, global kelp forests along temperate and polar coastlines are subjected to multiple threats and concurrent stressors (Butler et al., 2020; Smale et al., 2013). In response, significant efforts are being made to restore and counteract these declining kelp forests (Eger et al., 2022; Layton et al., 2020).

One particularly important seaweed in temperate parts of both hemispheres is the giant kelp, *Macrocystis pyrifera* (order Laminariales). It is a large (<35 m tall) species along the temperate coasts of southern Australasia, the northeast Pacific, and western South Africa (Bolton, 2010; Graham et al., 2007). Healthy kelp populations are characterized by fast sporophyte growth and large standing stock, which are linked to reproductive output and elevated zoospore concentrations; conversely, diminished zoospore concentrations may signal a declining population (Harley et al., 2012). Kelp forest ecosystems have dynamic patterns of decline and recolonization, characterized by periodic fluctuations typically spanning 3–5‐year cycles (Dayton et al., 1984, 1992; Ebeling et al., 1985; Graham et al., 1997). Recently, substantial multi‐decadal declines in kelp forest coverage have been documented throughout its geographic distribution, beyond the previously observed short‐term cycles (Krumhansl et al., 2016). Consequently, these prolonged ecosystem transformations have substantial cascading ecological implications, particularly for species dependent on kelp forest habitat (Edgar et al., 2023). These declines have been particularly profound in southeastern Australia, with over 90% canopy loss (Butler et al., 2020; Port et al., 2016). Therefore, efforts are being directed to restore giant kelp forests (e.g., Eger et al., 2022; Layton et al., 2020). Furthermore, *M. pyrifera* is harvested from wild populations and farmed, predominantly for alginates, liquid fertilizer, and abalone feed (Purcell‐Meyerink et al., 2021). For successful restoration and cultivation, a detailed understanding of the reproductive cycle is essential for sourcing seedstock for onward cultivation and restoration efforts, as well as monitoring the health of kelp populations locally (Hurd et al., 2023; Veenhof et al., 2023).

The life history of *Macrocystis pyrifera* is a heteromorphic alternation of generations between the diploid macroscopic sporophyte that produces haploid microscopic zoospores that are released into the water column, settle onto suitable substrata, and germinate into the haploid microscopic gametophyte. It is perennial, and its reproductive phenology is strongly controlled by local environmental conditions such as temperature, photoperiod, and wave action (Graham et al., 2007; Harley et al., 2012; Hurd et al., 2014). Mature sporophytes produce sporangia within sori. Sori form on specialized blades, called sporophylls, which are typically basal, but sporangia have also been observed on pneumatocyst‐bearing blades and apical meristem blades in New Zealand, Australia, and California (Graham et al., 2007; Leal et al., 2014; Leal et al., 2021; Visch pers. obs). Sori are characterized by being darker than nonreproductive tissue (Leal et al., 2021). The reproductive capacity of kelps can be related to the health and size of an individual (Geange, 2014; Mohring et al., 2013). Up to 10 million zoospores per cm^2^ tissue (Leal et al., 2014) of very small (width of 3 μm and length of up to 7 μm) motile zoospores are released from fertile sorus tissue and are then significantly diluted in the water column (Graham, 1999; Henry & Cole, 1982). Zoospores settle onto suitable substrate (e.g., rock) and develop into microscopic gametophytes that produce gametes that fertilize and eventually develop into juvenile sporophytes. Zoospores are mostly undetectable in seawater other than by microscopic analysis and are practically indistinguishable by species (Graham, 1999; Henry & Cole, 1982). Graham (1999) reported an average zoospore concentration for *M. pyrifera* of 7.7 zoospores · mL^−1^ using the light‐absorbance characteristics of plastids present in kelp zoospores from in situ plankton samples, with relatively high variation across sampling periods. To the best of our knowledge, this is currently the most accurate estimate of kelp zoospore concentrations in situ.

In the field, kelp reproduction is typically monitored by visual observation of sporophylls and sori tissue on mature individuals (e.g., Kaminsky et al., 2023; Roleda, 2016; Schaffelke et al., 2005). Visual observation requires diving, which is time and cost intensive, has associated safety risks, and has the potential for observer bias (Neushul, 1963). Consequently, these methodological limitations have been demonstrated to restrict both the spatial and temporal resolution of sampling reproduction across diverse marine taxa, not only kelp populations, but also red seaweeds, sharks, and tropical fish (Boussarie et al., 2018; Polanco Fernández et al., 2021; Saunders, 2005). The ability to track the reproductive cycle of *Macrocystis pyrifera*, as well as other seaweed species that exhibit heteromorphic alternation of generations, is therefore an area for improvement and an opportunity to deepen our understanding of reproductive phenologies.

Tracking of the reproductive cycle of kelps may be possible using molecular methods involving environmental DNA (eDNA) and quantitative polymerase chain reaction (qPCR). Environmental DNA is the use of water, soil, or other environment‐sourced samples to test for traces of DNA; this might be free‐floating DNA, shed cells, dissolved or particulate organic carbon, excrement, or other sources (Mathieu et al., 2020; Zeng et al., 2024). Environmental DNA samples have been used to detect a species within entire communities (Jeunen et al., 2019; Mathieu et al., 2020; Port et al., 2016) and to detect the presence of a single species that may be elusive (Spear et al., 2015; Takahashi et al., 2018), endangered, or invasive (Goldberg et al., 2013; Miyaguchi et al., 2008; Muha et al., 2019). To amplify and detect species within the sample, specific primers are required for qPCR (Piñol et al., 2019). A potential limitation of eDNA for tracking reproductive cycles is the inability to differentiate life stages contributing to the eDNA signal. This has been explored for various marine organisms, but not for seaweeds. For instance, Macquarie perch sperm DNA was isolated by targeting nuclear DNA, relying on distinct DNA content in different life stages (Bylemans et al., 2017). Scallop DNA size‐fractionation successfully isolated gamete‐sourced DNA but may not apply universally (Bayer et al., 2019). Ex situ experiments that isolated life stages and measured resulting eDNA signal (qPCR) have provided valuable insights into eDNA sample interpretation, such as the absence of detectable eDNA from eel eggs (Takeuchi et al., 2019). Similarly, exploration of life‐stage eDNA signal from crabs observed higher eDNA exudence at soft‐shell stages (Crane et al., 2021). Understanding these dynamics will enhance eDNA sample interpretation and guide experimental design. It has been suggested that the reproductive cycling of natural kelp populations can be tracked using eDNA (Nagasato et al., 2020). However, distinguishing the effect of microscopic zoospores and adult sporophyte tissue on seawater eDNA signal has not been tested.

Here, we assess the sensitivity of eDNA for detecting and resolving signals from zoospores and sporophytes of giant kelp *Macrocystis pyrifera* in a laboratory setting. We hypothesize that increases in eDNA signal can be attributed to increased zoospore concentration rather than the presence of sporophyte material. The results provide important information that will help improve the monitoring of reproductive cycles of seaweeds and *M. pyrifera* specifically.

## MATERIALS AND METHODS

### Primer design

The PCR primers were designed using mitochondrial DNA sequences of Tasmanian seaweed populations (Durrant et al., 2015). Haplotypes were aligned using MEGA X (Kumar et al., 2018; Stecher et al., 2020), allowing analysis of interspecies base mismatches and intraspecies sequence conservation of *Macrocystis pyrifera* and other locally sourced and dominant brown seaweeds: *Lessonia corrugata*, *Ecklonia radiata* (order Laminariales), and *Phyllospora comosa* (order Fucales). Primer3 was used to produce potential primer pairs (Untergasser et al., 2012; Koressaar & Remm, 2007). Primers were checked for species specificity on NCBI Nucleotide Blast.

### Primer validation

#### PCR specificity testing

Each primer pair (Table S1) was tested for species specificity in conventional PCR with genomic DNA from *Macrocystis pyrifera*, *Lessonia corrugata*, *Ecklonia radiata*, and *Phyllospora comosa*. Reactions consisted of 0.5 μM each forward and reverse primer, 10 μL MyTaq DNA Polymerase mix (Bioline), and 1 μL genomic DNA in a 20 μL reaction volume. A gradient PCR (annealing at 57–67°C) determined the cycling parameters that amplified the target without nontarget amplification (Appendix S1: Figure S1): 1 min at 95°C, 34 cycles of 20 s at 95°C, 20 s at 65°C, and 45 s at 72°C, followed by 1 min at 72°C. The primer pair mpy_F3_166/mpy_R3_166 was selected based on target species (*M. pyrifera*) fidelity.

#### qPCR optimization

Gametophyte DNA was extracted from existing *Macrocystis pyrifera* gametophyte cultures using the Qiagen Plant Mini Kit, as per Nagasato et al. (2020). The PCR products produced from this DNA, following the methods above, were purified using the Qiaquick PCR Purification Kit (Qiagen) and quantified by fluorometer. The PCR product was exponentially diluted 10^7^‐10^1^ copies · μL^− 1^, used as qPCR standards to optimize cycling parameters and determine assay sensitivity. The qPCR reactions were prepared in a Myra Liquid Handling Robot (Bio Molecular Systems) and run on a Qiagen RotorgeneQ.

### Dilution and life‐stage distinction experiments

#### Sample collection and preparation

*Macrocystis pyrifera* samples (16 nonreproductive sporophyte blades and two reproductive sporophylls) were collected from Blackmans Bay, Tasmania, Australia (43.0074° S, 147.3290° E), in July 2022. All appeared healthy with minimal biofouling. They were transported in a cool box containing seawater to a laboratory 20 min away and processed immediately. Nonreproductive sporophyte blades were wiped cleaned with paper towel and cut into segments from approximately halfway up the blade. For the “Life‐Stage Distinction Experiment,” eight 1.8 g wet weight pieces were cut. For the “Dilution Experiment,” eight pieces reducing in size were also cut for immediate use (see below), with cut edges wiped with paper towel.

Zoospores were released from sorus tissue that was cut from reproductive sporophylls, wiped clean with paper towels, and further washed with filtered (0.22 μm pore size) autoclaved seawater solution (from now on referred to as “autoclaved seawater”) three times before being placed between paper towels in a ziplock bag in a cool room (12°C) to desiccate overnight (Biancacci et al., 2022; Schwoerbel et al., 2022; Visch et al., 2023). Zoospores were released the next day by wiping the reproductive sporophylls with a paper towel to remove any prematurely released dead zoospores and placed in a beaker with autoclaved seawater (12°C) for ~1 h (Alsuwaiyan et al., 2019). Zoospore density was then determined using a hemocytometer.

#### Dilution experiments

A dilution experiment was performed to determine the individual effects of zoospore concentration and sporophyte biomass present in seawater on eDNA signal. The prepared nonreproductive sporophyte tissue pieces prepared above were immediately put into six jars containing 1 L autoclaved seawater. All jars were kept at 12°C, under constant gentle air flow with 0.2‐μm filters, and in constant light conditions (~80 μmol photons · m^−2^ · s^−1^). These conditions were close to ambient conditions in situ and have been shown to be favorable for zoospore survival and overall sporophyte health (Biancacci et al., 2022). The nonreproductive sporophyte tissues were 1.8, 0.9, 0.45, 0.225, and 0.1125 g wet weight, and jars with no sporophyte tissue served as blank controls. All treatments were replicated four times. Seawater from jars containing nonreproductive sporophyte tissue was sampled and filtered 24 h after adding the tissue. Additionally, experimental zoospore dilutions of 10,000, 1000, 100, and 10 zoospores · mL^−1^ were prepared in 1 L of autoclaved seawater. The jars with zoospores were sampled and filtered 4 h after zoospore addition, alongside blank controls. This time difference between sporophyte (24 h) and zoospore (4 h) treatments aimed to simulate a realistic ecological scenario with continuous sporophyte presence and zoospore release.

#### Life‐stage distinction experiment

This experiment tested four replicates of three treatments and a blank control: (1) sporophyte only (1.8 g wet weight), (2) zoospores only (100 zoospores · mL^−1^), (3) sporophyte + zoospores (1.8 g wet weight, and 100 zoospores · mL^−1^, respectively), and (4) a blank control. The nonreproductive sporophyte tissue pieces were prepared as outlined above and immediately placed into 2‐L plastic experimental jars containing 1 L of autoclaved seawater after being cleaned and maintained under the same experimental conditions as described above. The zoospores (100 zoospores · mL^−1^) were added to jars representing the “sporophyte + zoospore” and “zoospore only” treatment. To simulate ecologically relevant hydrodynamic conditions, in addition to continuous gentle aeration, the jars were occasionally agitated by swirling throughout the experiment. The eDNA sampling of “sporophyte” was performed 24 h after sporophyte addition and another 4 h later for all other treatments.

#### eDNA sampling

Every jar was mixed by swirling three times. From each jar, a 200‐mL seawater sample was aseptically collected and syringe‐filtered through a sterile 0.45‐μm cellulose nitrate membrane filter (47 mm diameter, Whatman, GF/F). Filters were placed into individual 15‐mL sterile tubes and frozen immediately at −20°C until extraction of DNA approximately 1 month post experiment.

#### DNA extraction, primer design, and validation for qPCR

DNA was extracted from filters using the Qiagen DNeasy PowerWater kit, following the guidelines with a 50 μL elution volume. Triplicate qPCR reactions (for samples, standards, and a No Template Control, NTC) were prepared using the Myra Robot and run on the Qiagen RotorgeneQ.

The qPCR cycling parameters were 2 min hold at 95°C, followed by 40 cycles of 5 s at 95°C and 20 s at 65°C, followed by a melt from 72°C to 95°C. Ten microliters of qPCR contained 5 μL Bioline SensiFast, 1 μL template DNA, 0.5 μM each forward and reverse primers. One qPCR run was conducted for samples of the “Life‐Stage Distinction Experiment,” and one for the “Dilution Experiment.”

### Data analysis

All plots and statistical analyses were performed using R software (R Core Team, 2023). Data outputs from qPCR in the form of quantification‐cycle values (Cq‐value, cycles required for amplified DNA to be detected) and measured DNA copies · μL^−1^ were used to determine success of the sample run and for subsequent analysis. The data from both the “Dilution Experiment” and “Life‐Stage Distinction Experiment” were adjusted to account for contamination in the no template controls (NTCs; containing all the qPCR reagents except for the target DNA) by subtracting from all other samples the mean NTC‐value. Prior to statistical analysis, data were checked for violations of analysis of variance (ANOVA) assumptions (heteroscedasticity and normality of residuals), formally tested using the Shapiro–Wilk test (α = 0.05) and transformed when required.

For the “Dilution Experiment,” a Spearman's rank correlation test between DNA concentration and zoospore concentration and between DNA concentration and sporophyte tissue wet weight were performed with the function cor.test(). As the data for the “Life‐Stage Distinction Experiment” violated assumptions for a parametric test, a non‐parametric Kruskal–Wallis rank‐sum test was performed using the function kruskal. test(). When the main effects were significant (α = 0.05), a Dunn's post hoc test was used to determine the nature of differences between treatments using the function dunnTest() from the package FSA (Ogle et al., 2022).

To determine at what concentrations zoospores are expected to contribute to the eDNA signal beyond the sporophyte, values obtained from the dilution experiments were also subjected to linear regression (lm function), the results graphically represented using the confint and geom_smooth functions, and the eDNA signal above which zoospores would predominate was identified.

## RESULTS

### Primer validation

The primer pairs (mpy_F3_166/mpy_R3_166) cross‐amplified between *Macrocystis pyrifera* and *Ecklonia radiata* at some temperatures, but at a higher temperature (65°C), we were able to amplify *M. pyrifera* and not *E. radiata* (Figure S1). Hence, primers mpy_F3_166/mpy_R3_166 were used with a 65°C annealing temperature for all subsequent assays.

### Dilution experiments

**FIGURE 1:**
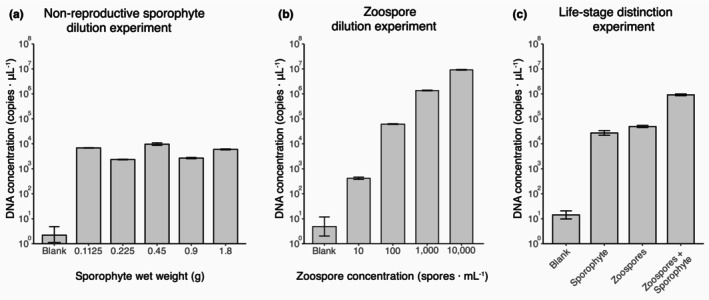
Mean DNA concentrations (copies · μL^−1^) of *Macrocystis pyrifera* of (a) the nonreproductive sporophyte dilution experiment, (b) the zoospores dilution experiment, and (c) the life‐stage distinction experiment where the zoospore concentration was 100 zoospores · mL^−1^ and the sporophyte wet weight 1.8 g, and both combined to create the zoospore + sporophyte treatment. Error bars represent standard error (*SE*), with *n* = 4.

Copies of amplified DNA were detected at all zoospore concentrations and sporophyte wet weights, including blank control treatments (Figure 1a,b). Zoospore concentration and DNA concentration (NTC‐adjusted) were significantly positively correlated (Spearman's rank ρ‐value = 0.982; *p* < 0.001; Figure 1a). Conversely, no significant correlations were observed between sporophyte wet weight and either DNA concentration (Spearman's rank ρ‐value = 0.367; *p* = 0.134; Figure 1b) or Cq‐values (Spearman's rank ρ‐value = −0.367; *p* = 0.134; Appendix S1: Figure S2).

#### Life‐stage distinction experiment

DNA concentration (χ^2^ = 35.92, *df* = 3, *p* < 0.001) and Cq‐values (χ^2^ = 35.66, *df* = 3, *p* < 0.001) differed significantly between treatments. Subsequent pairwise Dunn's test indicated a significant difference in the DNA concentration for all comparisons except individual zoospore (100 zoospores · mL^−1^) and sporophyte (1.8 g wet weight; Table 1). Comparisons of Cq‐values mirrored those of DNA concentration, except the sporophyte + zoospore and zoospore comparison was also nonsignificant (Appendix S1: Figure S2 and Table S2).

**TABLE 1:** Pairwise comparisons of the DNA concentration of *Macrocystis pyrifera* between treatment groups of the life‐stage distinction experiment using Dunn's test.

Group comparisons	Mean difference	*p*‐value
Blank versus Sporophytes	−2.45	**0.043**
Blank versus Zoospores	−3.25	**0.003**
Blank versus Zoospores + Sporophytes	−5.96	**<0.001**
Sporophyte versus Zoospores	−0.87	1.000
Sporophytes + Zoospores versus Sporophytes	3.79	**<0.001**
Sporophytes + Zoospores versus Zoospores	2.92	**0.010**

*Note*: *p* < 0.05 is indicated in bold.

#### Applied and ecological context

**FIGURE 2:**
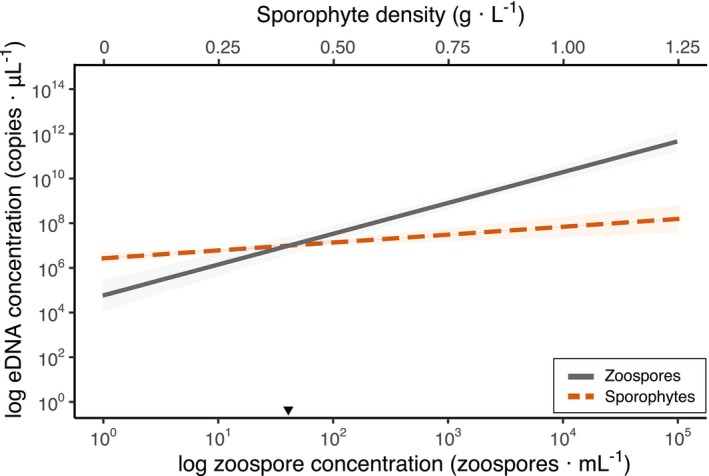
Modeled eDNA concentrations of *Macrocystis pyrifera* in the zoospore dilution experiment (gray) and detected DNA concentration during the sporophyte dilution experiment (dashed red) with their respective 95% confidence interval. Note, the log concentration of zoospores on the primary *x*‐axis, and the sporophyte wet weight (g) per L seawater on the secondary *x*‐axis. The vertical dashed gray arrow highlights the intersection point of sporophyte and zoospore regression lines at ~37 zoospores · mL^−1^, indicating that zoospore concentrations above this value will produce eDNA signal attributed primarily to zoospores rather than sporophyte tissue.

Utilizing model‐derived values obtained from the zoospore and sporophyte dilution experiments, the point of intersection between both linear regression lines was approximately 37 zoospores · mL^−1^ (Figure 2). This finding implies that zoospore concentrations exceeding this threshold are predicted to elevate the eDNA signal above what could be purely attributed to sporophyte tissue.

## DISCUSSION

This controlled laboratory study specifically explored whether nearby, nonreproductive sporophyll tissue from a kelp can influence the eDNA signal in seawater of samples collected to detect seasonal variation in zoospore concentration. It highlights a substantial contribution of *Macrocystis pyrifera* nonreproductive sporophyte tissue to the total eDNA signal, even when only small amounts of tissue are present in seawater (e.g., 0.1125 g wet weight · L^−1^). However, there appears to be no significant correlation between the detected DNA concentration and sporophyte wet weight. In contrast, such a correlation exists for zoospore density, and from our experiment, it appears that zoospore densities above 37 zoospores · mL^−1^ will provide an eDNA signal greater than could be otherwise ascribed to sporophyte tissue. While the specificity of this assay to *M. pyrifera* requires further testing, its sensitivity appears adequate for detecting broad seasonal patterns of *M. pyrifera* reproduction, assuming zoospore densities are high at some periods, regardless of the adult sporophyte biomass growing nearby.

The combined results of the dilution experiment and the life‐stage distinction experiment indicate that relatively high zoospore concentrations significantly contribute to the detected eDNA signal. This confirms earlier results using a similar qPCR method in quantifying the reproductive phenology of two Laminarian kelps (*Undaria pinnatifida* and *Saccharina japonica*) in Japan (Nagasato et al., 2020). Over a 2‐year period, Nagasato et al. (2020) observed a constant DNA presence in seawater near natural kelp reefs of both species, corresponding to approximately 50 zoospores · mL^−1^ as a baseline, rising to approximately 200 zoospores · mL^−1^ during the reproductive season. Our results are consistent with these observations. We observed a strong correlation between increasing zoospore concentrations and the detected DNA concentration, indicating that changing zoospore densities significantly affect the eDNA signal.

Unlike zoospore concentration, our study revealed only a weak correlation between the sporophyte wet weight and DNA concentration. Surprisingly, even small amounts of sporophyte biomass contributed substantially to the eDNA signal, which remained relatively stable despite increases in sporophyte biomass. This contrasts with previous studies reporting positive correlations between eDNA content and biomass (including seaweed; Landenmark et al., 2015). Our findings challenge the use of eDNA as a reliable biomarker for sporophyte biomass estimation, which is particularly significant given that approximately 3% of cellular organic carbon is DNA (Holm‐Hansen, 1969). Recent studies have used eDNA signals as indicators of seaweed carbon sequestration capacity (Geraldi et al., 2019; Zeng et al., 2024). However, our results suggest this approach may require further validation. This is further compounded by the fact that in kelp, much cellular organic carbon exists as detritus from blade erosion, fragmentation, and frond dislodgement (Krumhansl & Scheibling, 2012). This may explain the unexpected stability of eDNA levels across varying sporophyte biomass in our study, where we used healthy tissue samples. Although our findings are a good starting point for future research, they emphasize the need for caution when using eDNA methods to estimate the demography and standing stock of *Macrocystis pyrifera* or other seaweed species.

*Macrocystis pyrifera* is a highly fecund species, exceeding 10^8^ zoospores · day^−1^ from sporophylls showing well‐developed sporangial areas (Anderson & North, 1966; Gaylord et al., 2006). Furthermore, fertile plants can be observed year round, and fecundity (both sporophyll biomass and zoospore production) is positively related to its total biomass (Geange, 2014; Reed, 1987). Variability in reproductive output leads to high temporal variability in zoospore concentrations (Graham, 1999). Zoospore dispersal is typically limited and highly variable due to stochastic effects of turbulence (Gaylord et al., 2006). Approximately 50% of zoospores disperse less than 100 m in the flow conditions common in *M. pyrifera* beds (Gaylord et al., 2002). Furthermore, reproductive sporophylls have been observed in free‐floating *Macrocystis* spp. rafts, indicating that zoospores can be dispersed at longer distances (up to 100s km‐scale) from the nearest *Macrocystis* spp. beds (Hernández‐Carmona et al., 2006; Layton et al., 2022; Macaya et al., 2005). The production and release of zoospores in *M. pyrifera* are synchronized (Reed et al., 1997) to increase settlement density and chances of subsequent fertilization involving gametophytes (Reed, 1990; Reed et al., 1991). This increases the likelihood of detectable variation in the eDNA signal from zoospores. However, zoospore concentrations significantly decrease with increasing distance from the nearest kelp population (Edwards, 2022). Therefore, when detecting reproductive patterns using eDNA, seawater should be collected as close as possible to a kelp population, even though zoospores may be present at much greater distances from the nearest reef.

Recently, advancements in eDNA technology have significantly enhanced research into marine biodiversity and have benefitted policy making (Zeng et al., 2024). Our study indicates eDNA approaches can be useful to investigate globally declining kelp forests, including habitat‐forming species such as *Macrocystis pyrifera* (Edgar et al., 2023; Krumhansl et al., 2016; Smale, 2020). Healthy kelp populations are characterized by high sporophyte growth, productivity, and biomass, which are positively correlated with reproductive output and thus elevated zoospore concentrations in the water column. Conversely, reduced zoospore concentrations may indicate a population in decline, of which the early signs may be captured by the eDNA method tested in the present study (Harley et al., 2012). In light of recent efforts to restore declining kelp forests (Edgar et al., 2023; Layton et al., 2020), the results of this research represent a significant step toward understanding the relationships between zoospores and sporophytes with eDNA, with a view to monitoring *M. pyrifera* reproduction. To improve its validity and sensitivity, future research could combine methods by linking light‐absorbance techniques (as per Graham, 1999) with eDNA methods (as per this study and Nagasato et al., 2020) of seawater samples collected periodically near kelp forests. These methods should be verified by field observations of reproductive tissue by SCUBA diving. Primers targeting different DNA types (organelle and nuclear DNA) should also be compared, along with assessments of eDNA decay rates through both space and time.

## CONCLUSIONS

This laboratory study has demonstrated the potential of eDNA as a valuable tool for tracking reproduction in the giant kelp *Macrocystis pyrifera*, with broader implications for assessing the overall health of targeted kelp populations. With refinement and additional research, clearer distinctions in eDNA life‐stage source may be defined, but validation of the results in the field is needed. The ability to monitor giant kelp reproduction through eDNA analysis offers significant advantages over currently applied methods that rely on SCUBA diving or snorkeling. This knowledge is important for informing conservation strategies, guiding restoration efforts, and selecting appropriate seedstock for aquaculture. These activities, which rely on the collection of reproductive material from wild kelp populations, will benefit greatly from an improved understanding of reproductive patterns at relatively large spatiotemporal scales provided by eDNA monitoring. Finally, this work has implications for other seaweed species for which tracking the reproductive phenology using eDNA can be used as an indicator for the overall health.

## AUTHOR CONTRIBUTIONS

**Madeline R. Ward:** Formal analysis (lead); methodology (lead); writing – original draft (lead). **Christopher P. Burridge:** Formal analysis (equal); methodology (equal); supervision (equal); writing – review and editing (equal). **Sharee McCammon:** Methodology (equal); writing – review and editing (equal). **Adam Smolenski:** Methodology (equal); writing – review and editing (equal). **Catriona L. Hurd:** Methodology (equal); supervision (equal); writing – review and editing (equal). **Wouter Visch:** Conceptualization (lead); data curation (equal); funding acquisition (lead); methodology (lead); project administration (lead); supervision (lead); writing – review and editing (lead).

## FUNDING INFORMATION

This research was funded by the “Grants‐in‐Aid of Research (GIAR) Program” funded by the Phycological Society of America.

## Supporting information


**Table S1.** *Macrocystis pyrifera* primer pair (mtDNA) descriptions designed for species‐specific amplification. Primer pair *mpy_F3_166/mpy_R3_166* (highlighted in gray) was selected for all subsequent experiments based on target species fidelity with an annealing temperature of 65°C (Figure S1).
**Figure S1.** Heat‐gradient PCR gel electrophoresis image. *Macrocystis pyrifera* and *Ecklonia radiata* DNA amplified with *mpy_F3_166/mpy_R3_166* primer pair. Temperate ranges from 57°C (blue/left) to 67°C (red/right). As the primer pair amplified *M. pyrifera* and not *E. radiata* at a 65°C annealing temperature, this temperature was used for all subsequent assays.
**Figure S2.** Barplots showing the mean quantification‐cycle values (Cq‐values) of *Macrocystis pyrifera* of (a) the non‐reproductive sporophyte dilution experiment, (b) the zoospore dilution series experiment, and (c) treatment effects of the life‐stage distinction experiment where the zoospore concentration was 100 zoospores · mL and the sporophyte wet weight 1.8 grams, and both combined to create the zoospore + sporophyte treatment. Error bars represent standard error (*SE*), with *n* = 4 in both dilution experiments and *n* = 12 in the life‐stage distinction experiment.
**Table S2.** Pairwise comparisons of the Cq values of *Macrocystis pyrifera* between treatment groups based of the life‐stage distinction experiment using Dunn's test. *p* < 0.05 are highlighted in bold.
