# Supplementary material for: Applications of environmental DNA monitoring for seaweed reproductive phenology: A case study with giant kelp (Macrocystis pyrifera)
Source: J Phycol. 2025 Mar 18;61(2):288–98. doi: 10.1111/jpy.70000 (PMC12044407; doi:10.1111/jpy.70000)
Supplement: Supplementary file 1 — Table S1. Macrocystis pyrifera primer pair (mtDNA) descriptions designed for species‐specific amplification. Primer pair mpy_F3_166/mpy_R3_166 (highlighted in gray) was selected for all subsequent experiments based on target species fidelity with an annealing temperature of 65°C (Figure S1). Figure S1. Heat‐gradient PCR gel electrophoresis image. Macrocystis pyrifera and Ecklonia radiata DNA amplified with mpy_F3_166/mpy_R3_166 primer pair. Temperate ranges from 57°C (blue/left) to 67°C (red/right). As the primer pair amplified M. pyrifera and not E. radiata at a 65°C annealing temperature, this temperature was used for all subsequent assays. Figure S2. Barplots showing the mean quantification‐cycle values (Cq‐values) of Macrocystis pyrifera of (a) the non‐reproductive sporophyte dilution experiment, (b) the zoospore dilution series experiment, and (c) treatment effects of the life‐stage distinction experiment where the zoospore concentration was 100 zoospores · mL and the sporophyte wet weight 1.8 grams, and both combined to create the zoospore + sporophyte treatment. Error bars represent standard error (SE), with n = 4 in both dilution experiments and n = 12 in the life‐stage distinction experiment. Table S2. Pairwise comparisons of the Cq values of Macrocystis pyrifera between treatment groups based of the life‐stage distinction experiment using Dunn's test. p < 0.05 are highlighted in bold. [file JPY-61-288-s001.docx]

**Supplementary material**

| **Table S1.** *Macrocystis pyrifera* primer pair (mtDNA) descriptions designed for species-specific amplification. Primer pair *mpy_F3_166/mpy_R3_166* (highlighted in gray) was selected for all subsequent experiments based on target species fidelity with an annealing temperature of 65°C (Figure S1). | | | | | | |
| --- | --- | --- | --- | --- | --- | --- |
| **#** | **Name (Species_**  **primer_productsize)** | **Direction** | **Start**  **position (nt)** | **Sequence (5' to 3')** | **Melting temp. (°C)** | **GC**  **content (%)** |
| 1 | mpy_F1_173 | Forward (L) | 230 | ggccctgaatgtgcttgaat | 58.81 | 50.00 |
|  | mpy_R1_173 | Reverse (R) | 402 | cctctaatgattggtgcccc | 58.01 | 55.00 |
| 2 | mpy_F2_176 | Forward (L) | 170 | gcccctaaaatagaagcagca | 58.35 | 47.62 |
|  | mpy_R2_176 | Reverse (R) | 345 | tggttgttgcctccttcttt | 57.55 | 45.00 |
| 3 | mpy_F3_166 | Forward (L) | 108 | gcaacctatccattgtcatacca | 58.54 | 43.48 |
|  | mpy_R3_166 | Reverse (R) | 273 | acagtatacccaccgcttagt | 58.26 | 47.62 |


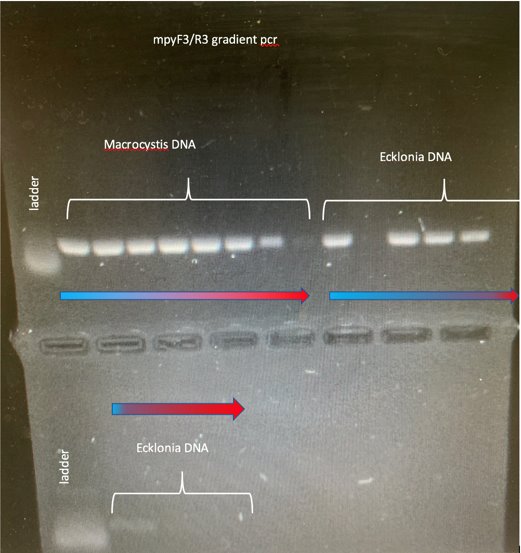


**Figure S1.** Heat-gradient PCR gel electrophoresis image. *Macrocystis pyrifera* and *Ecklonia radiata* DNA amplified with *mpy_F3_166/mpy_R3_166* primer pair. Temperate ranges from 57 °C (blue/left) to 67 °C (red/right). As the primer pair amplified *M. pyrifera* and not *E. radiata* at a 65°C annealing temperature, this temperature was used for all subsequent assays.

**Figure S2**. Barplots showing the mean quantification-cycle values (Cq-values) of *Macrocystis pyrifera* of (A) the non-reproductive sporophyte dilution experiment, (B) the zoospore dilution series experiment, and (C) treatment effects of the life-stage distinction experiment where the zoospore concentration was 100 zoospores/mL and the sporophyte wet weight 1.8 grams, and both combined to create the zoospore + sporophyte treatment. Error bars represent standard error (SE), with *n* = 4 in both dilution experiments and *n* = 12 in the life-stage distinction experiment.

| **Table S2.** Pairwise comparisons of the Cq values of *Macrocystis pyrifera* between treatment groups based of the life-stage distinction experiment using Dunn’s test. *p* < 0.05 are highlighted in bold. | | |
| --- | --- | --- |
| Group comparisons | Mean difference | *p*-value |
| Blank vs Sporophytes | 2.27 | 0.069 |
| Blank vs Zoospores | 3.61 | **<0.001** |
| Blank vs Zoospores + Sporophytes | 5.77 | **<0.001** |
| Sporophyte vs Zoospores | 1.44 | 0.445 |
| Sporophytes + Zoospores vs Sporophytes | -3.78 | **<0.001** |
| Sporophytes + Zoospores vs Zoospores | -2.33 | 0.059 |
